# Measuring case severity: a novel tool for benchmarking and clinical documentation improvement

**DOI:** 10.1186/s12913-022-07935-1

**Published:** 2022-04-15

**Authors:** Jie Xiang, Paul W. Durance, Louisa C. Griffes, Yalei Chen, Rishi R. Bakshi

**Affiliations:** 1 Revenue Cycle Department, Michigan Medicine, University of Michigan Health, Ann Arbor, MI 48105 USA; 2 Domino’s Pizza, Inc, Ann Arbor, MI USA; 3 Financial Services Department, Michigan Medicine, University of Michigan Health, Ann Arbor, MI USA; 4 Quality Analytics, Quality Department, Michigan Medicine, University of Michigan Health, Ann Arbor, MI USA; 5 Department of Public Health Sciences, Henry Ford Health System, Detroit, MI USA; 6 Departments of Physical Medicine & Rehabilitation and Revenue Cycle, Michigan Medicine, University of Michigan Health, Ann Arbor, MI USA

**Keywords:** Severity of Illness, APR DRG, Body Systems, Elixhauser Comorbidities, J Scores, Benchmark, Clinical Documentation Improvement

## Abstract

**Background:**

Severity of illness (SOI) is an All Patients Refined Diagnosis Related Groups (APR DRG) modifier based on comorbidity capture. Tracking SOI helps hospitals improve performance and resource distribution. Furthermore, benchmarking SOI plays a key role in Quality Improvement (QI) efforts such as Clinical Documentation Improvement (CDI) programs. The current SOI system highly relies on the 3 M APR DRG grouper that is updated annually, making it difficult to track severity longitudinally and benchmark against hospitals with different patient populations. Here, we describe an alternative SOI scoring system that is grouper-independent and that can be tracked longitudinally.

**Methods:**

Admission data for 2019–2020 U.S. News and World Report Honor Roll facilities were downloaded from the Vizient Clinical Database and split into training and testing datasets. Elixhauser comorbidities, body systems developed from the Healthcare Cost and Utilization Project (HCUP), and ICD-10-CM complication and comorbidity (CC/MCC) indicators were selected as the predictors for orthogonal polynomial regression models to predict patients’ admission and discharge SOI. Receiver operating characteristic (ROC) and Precision-Recall (PR) analysis, and prediction accuracy were used to evaluate model performance.

**Results:**

In the training dataset, the full model including both Elixhauser comorbidities and body system CC/MCC indicators had the highest ROC AUC, PR AUC and predication accuracy for both admission (ROC AUC: 92.9%; PR AUC: 91.0%; prediction accuracy: 85.4%) and discharge SOI (ROC AUC: 93.6%; PR AUC: 92.8%; prediction accuracy: 86.2%). The model including only body system CC/MCC indicators had similar performance for admission (ROC AUC: 92.4%; PR AUC: 90.4%; prediction accuracy: 84.8%) and discharge SOI (ROC AUC: 93.1%; PR AUC: 92.2%; prediction accuracy: 85.6%) as the full model. The model including only Elixhauser comorbidities exhibited the lowest performance. Similarly, in the validation dataset, the prediction accuracy was 86.2% for the full model, 85.6% for the body system model, and 79.3% for the comorbidity model. With fewer variables and less model complexity, the body system model was more efficient and was determined to be the optimal model. The probabilities generated from this model, named J_Score and J_Score_POA, successfully measured SOI and had practical applications in assessment of CDI performance.

**Conclusions:**

The J_Scores generated from the body system model have significant value in evaluating admission and discharge severity of illness. We believe that this new scoring system will provide a useful tool for healthcare institutions to benchmark patients’ illness severity and augment Quality Improvement (QI) efforts.

**Supplementary Information:**

The online version contains supplementary material available at 10.1186/s12913-022-07935-1.

## Background

Healthcare institutions increasingly emphasize improved outcomes and performance and focus efforts to improve quality of care and patient safety while lowering costs [1, 2]. They cannot determine whether their efforts are satisfactory without tracking outcomes and comparing with peers, so benchmarking is widely applied within healthcare organizations to improve their clinical performance and management of operations [3–5]. Hospitals have also started Clinical Documentation Improvement (CDI) programs to improve documentation quality; these programs ensure better patient outcomes, optimized data quality and accurate reimbursement [6, 7].

Vizient is the largest member-driven health care performance improvement company in the U.S. and it provides services to about 95% of the nation’s academic medical centers and more than 50% of the nation’s acute care health systems [8]. Using data collected by Vizient, members can benchmark many key performance indicators such as Case Mix Index (CMI), Length of Stay (LOS), Expected Risk of Mortality (EROM), and Severity of Illness (SOI) [9–12]. SOI describes the disease severity in hospitalized patients and measures the physical effects of disease on a patient. It is a powerful tool to track resource consumption and to track patient outcomes. In addition, SOI is also closely related to cost, revenue, and profit [13]. The admission and discharge SOI are created by the 3 M coding algorithm. The admission SOI is important for hospitals to measure the health status and severity of illness of a patient when he/she is admitted. Hospitals can use admission SOI to estimate resource distribution. Discharge SOI can be used for prospective payment and risk adjustment in quality reporting. Benchmarking SOI helps hospitals better evaluate their clinical performance and distribution of resources by comparing them to peers. The SOI levels presented in Vizient data come from the All Patients Refined Diagnosis Related Groups (APR DRG) classification system developed by 3M [14, 15] Each APR DRG has four categorical severity levels: minor, moderate, major and extreme. The SOI subclasses are related to the APR DRG grouper that is updated annually by 3M. However, there are several limitations using the current SOI system. Firstly, cross-category comparison of disease severity is less meaningful. The same SOI level from different APR DRG does not mean the same level of disease burden. Secondly, it is hard to compare severity among institutions because of each institution’s unique patient mix. Lastly, it is difficult to track yearly clinical performance using longitudinal data, given that the grouper is updated annually. Therefore, we sought to develop a novel measure of SOI that is grouper independent.

To find the appropriate predictors, we compared several models targeting Elixhauser comorbidities, body systems for chronic condition indicators, and complication or comorbidity (CC) or major complication or comorbidity indicators (MCC) [16–19]. The Elixhauser comorbidities are a comprehensive set of measures to identify different pre-existing conditions based on secondary diagnoses listed in hospital administrative data. The system was developed by Anne Elixhauser using all adult, nonmaternal inpatients from acute care hospitals in California in 1992 [20]. It includes 30 comorbidity measures that are associated with considerable increases in LOS, hospital charges, and mortality. The comorbidities are usually not directly related to the primary reason for the inpatient stay, but they have a possible effect on outcomes used to assess the quality of care. The Agency for Healthcare Research and Quality (AHRQ) has created a powerful Healthcare Cost and Utilization Project (HCUP) tool called Elixhauser Comorbidity Software Refined for ICD-10-CM, which can be applied to ICD-10 diagnosis codes to create a comorbidity profile [16, 17]. AHRQ also created another tool to categorize ICD-10-CM codes into 18 body systems [19]. Body systems allow us to correct for regional differences in patient mix and comorbidity-driven DRG modifiers, so that we can compare the intensity of severity that is independent on types of diseases. These tools provide the potential indicators for predicting the severity of illness. Another valuable resource is the list of all of the ICD-10 codes that are defined as either a CC or MCC diagnoses, released by Center for Medicare & Medicaid Services (CMS) [21]. We combined CC/MCC levels with body systems and created a 3-level indicator for each body system, indicating whether the body system has any CC or MCC diagnosis code. In this study, the Elixhauser comorbidities and 18 body systems with CC/MCC indicators were used as predictors to evaluate case severity. Instead of predicting four categorized SOI levels, we aimed to develop a model to better predict high and low illness severity that is independent of APR DRG assignment. Additionally, the probabilities generated from the model would be used as a quantitative measurement of SOI.

## Methods

### Data Sources

This study included 923,266 inpatient cases discharged between July 1^st^, 2018 and June 30^th^, 2019 from the 2019–2020 U.S. News and World Report Best Hospitals Honor Roll [22]. All cases used the same version of APR DRG. This study included adult inpatients with age >  = 18 (including maternity). Hospital encounters classified as inpatient status were included. Observation, Emergency Department, and Outpatient encounters were not included. Patients may be admitted multiple times; each hospital admission is a separate encounter in the study. Patients’ clinical data were downloaded from Vizient Clinical Data Base (CDB). Body system categories and Elixhauser Comorbidity indicators came from HCUP [16, 19]. The list of CC/MCC diagnosis codes was downloaded from the FY2019 CMS final rule and correction notice data files [21, 23]. Information related to case review and query by clinical documentation specialists came from an internal database within our institution. All the data used in this study were deidentified before analysis in accordance with HIPPA guidelines.

### Independent Variables and Outcome

In this paper, we predicted both admission and discharge SOI. All the diagnosis codes from a patient were used to predict the SOI at discharge, and only the present-on-admission (POA) and exempt diagnosis codes were used to predict the SOI on admission [24].

#### Body system CC/MCC level indicators

ICD-10 diagnosis codes were grouped into 18 body systems using HCUP software [19]. We combined the body system indicators and CC/MCC levels to create 18 variables. Each ordinal variable had three severity levels: 0, 1, and 2. Level 0 represented no diagnosis code in the specific body system. Level 1 represented there were one or more diagnosis codes in the body system, but none of them were CC or MCC codes. Level 2 indicated there was at least one CC or MCC diagnosis code in the body system.

#### Elixhauser comorbidity indicators

Elixhauser comorbidities were generated from Elixhauser comorbidity software v2019.2 (beta version) and SAS analysis program (COMOANALY_ICD10CM_2019_1.sas) that were downloaded from HCUP [17]. Each variable indicated whether the patient had a specific type of Elixhauser comorbidity based on the secondary diagnosis codes.

#### Outcome

APR DRG SOI minor and moderate levels (SOI = 1 or 2) were grouped into a low severity category, and major and extreme levels (SOI = 3 or 4) were considered as high severity. Our models predicted the probability of being a high SOI case, so, the outcome is a binary response variable. The probabilities calculated from the model were named J_Score_POA for admission SOI and J_Score for discharge SOI, which can be used to quantitatively measure the severity.

### Variable Selection and Model Validation

Data were randomly split into training and testing datasets in a ratio of three to one. Orthogonal polynomial regression was applied to the training dataset [25–28]. Three models were constructed and compared: the full model, the comorbidity model, and the body system model. The full model included all the Elixhauser comorbidities and 18 body systems with 3 levels of CC/MCC indicators (48 variables), the comorbidity model only included Elixhauser comorbidities (30 variables), and the body system model only involved body system CC/MCC indicators (18 variables). Receiver Operating Characteristic (ROC) and Precision-Recall (PR) analysis, together with prediction accuracy were used to measure the performance of the classification models [29, 30]. Prediction accuracy was identified as the percentage of correctly classified cases using the optimal cutoff points from ROC curves. Finally, models were applied to the independent testing dataset to predict the category of case severity using optimal cut-off points obtained from the training dataset. For admission SOI, the optimal cutoff points were 0.422 for the full model, 0.413 for the comorbidity model, and 0.411 for the body system model. For discharge SOI, the optimal cutoff points were 0.401 for the full model, 0.407 for the comorbidity model, and 0.412 for the body system model.

### Statistical analyses

Descriptive statistics (counts, mean, and proportions) were used to examine the similarity between training and testing datasets. The optimal cutoff point was defined as the value that minimizes the Euclidean distance between the ROC curve and the upper left corner of the graph using the training dataset. All the statistical analyses were performed in RStudio software (version 1.3.959). Statistical significance was defined as p < 0.05. No imputations were performed in the analysis.

## Results

### Characteristics of discharged cases

Nine hundred twenty-three thousand two hundred sixty-six discharged cases extracted from the Vizient Clinical Data Base were randomly split into training data (692,450 cases) and testing data (230,816 cases). We first compared the patients’ demographic and clinical information (Table 1). The mean age of patients from two datasets was 49 years old. Around 53% of patients were female, and the distribution of race categories was also very close (63% White, 15% Black, 5% Asian, and 11% other race). Patients in both data sets also had comparable admit and discharge APR DRG SOI levels.

**Table 1:** Distribution of Patients’ demographic characteristics and severity of illness for training dataset and testing dataset

	**Level**	**Training Data**	**Testing Data**	**p-value**
Age	Mean	49.09	49.01	0.22
Sex	Male	322,458 (46.57%)	107,758 (46.69%)	0.58
	Female	369,974 (53.43%)	123,051 (53.31%)	
Race	White	433,941 (62.67%)	144,910 (62.78%)	0.09
	Black	101,392 (14.64%)	33,795 (14.64%)	
	Asian	36,688 (5.30%)	11,860 (5.14%)	
	Other	73,422 (10.60%)	24,432 (10.60%)	
	Unavailable	33,505 (4.84%)	11,308 (4.90%)	
	Declined	11,834 (1.71%)	3975 (1.71%)	
	Unknown	1668 (0.24%)	536 (0.23%)	
Discharge APR-DRG SOI	1	161,149 (23.27%)	53,824 (23.32%)	0.39
	2	227,454 (32.85%)	75,710 (32.80%)	
	3	205,154 (29.63%)	68,098 (29.50%)	
	4	98,693 (14.25%)	33,184 (14.38%)	
Admit APR-DRG SOI	1	177,235 (25.60%)	59,225 (25.66%)	0.53
	2	238,968 (34.51%)	79,329 (34.37%)	
	3	198,686 (28.69%)	66,228 (28.69%)	
	4	77,561 (11.20%)	26,034 (11.28%)	

We then further looked at patients’ Elixhauser comorbidities and the MS-DRG complication and comorbidity (CC or MCC) diagnosis indicators in Clinical Classifications Software Refined (CCSR) body systems (Supplementary table 1). Patients in the two datasets had similar proportions of the 30 Elixhauser comorbidities and three CC/MCC levels in 18 body systems. Hypertension (HTN) and fluid, electrolyte disorders (LYTES), deficiency anemias (ANEMDEF) and chronic pulmonary disease (CHRNLUNG) were the top 4 comorbidities observed in patients: about 26% of patients with HTN, 24% with LYTES, 18% with ANEMDEF, and 17% with CHRNLUNG. Body systems 3 (Endocrine; nutritional; and metabolic diseases and immunity disorders), 7 (Diseases of the circulatory system) and 10 (Diseases of the genitourinary system) had the greatest number of patients with CC or MCC diagnosis codes. Overall, the training data and testing data samples were similar in both demographic and clinical characteristics.

### J_Scores generated from the body system model were reliable for predicting case severity

**Fig. 1:**
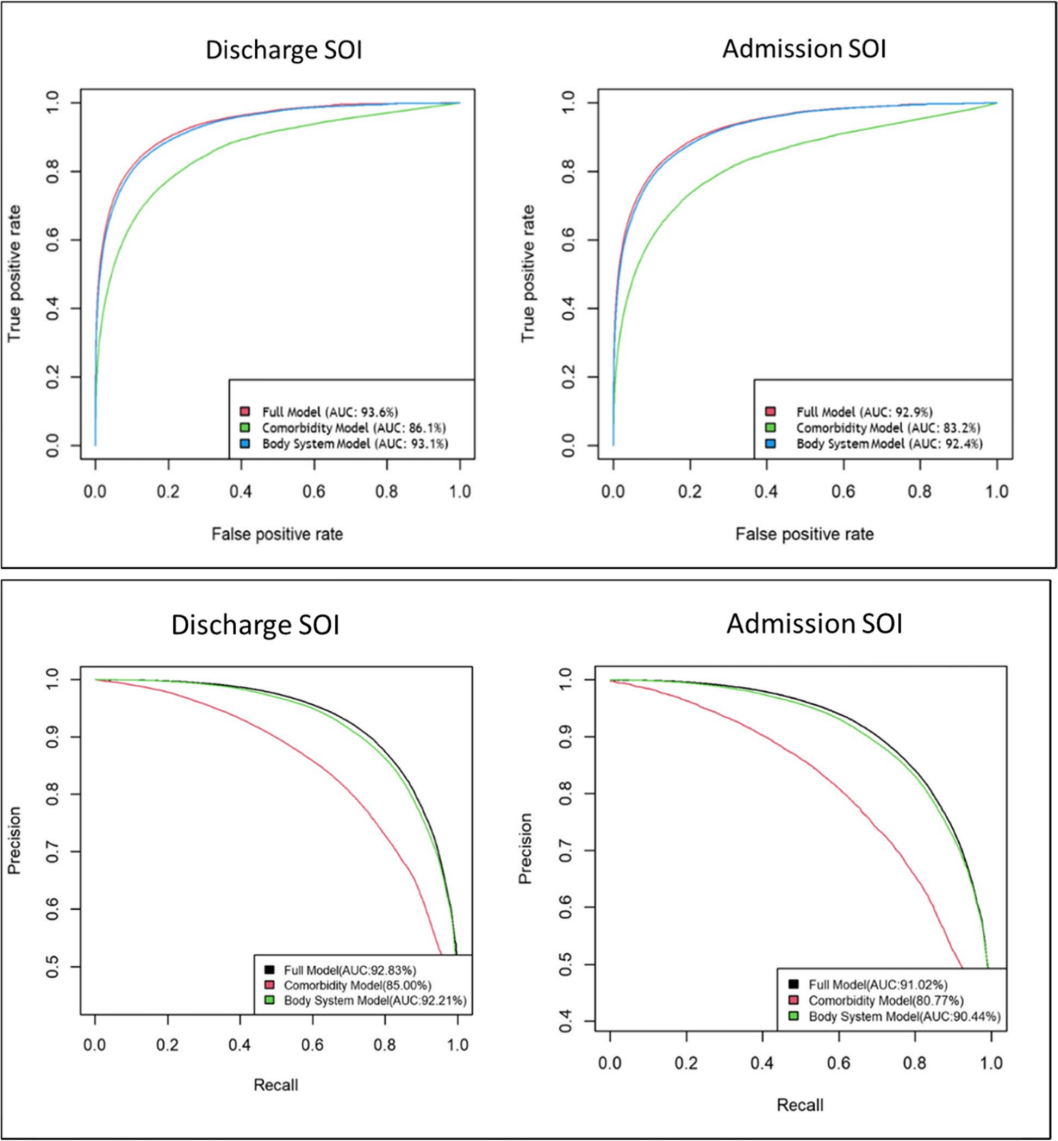
ROC curves and Precision-Recall curves for regression models predicting admission and discharge SOI

We applied three orthogonal polynomial regression models to the training data to predict the severity levels of each case. The prediction of admission and discharge SOI used the same algorithm; they differ based on diagnoses present on admission and discharge, respectively. To evaluate the performance, we examined ROC curves and PR curves. As shown in Fig. 1, when using all the diagnosis codes to map the Elixhauser comorbidities and body system CC/MCC indicators in discharge SOI models, the full model and body system model demonstrated similar AUC scores in both ROC curves (93.6% and 93.1%) and PR curves (92.8% and 92.2%). The comorbidity model had the lowest AUC (ROC: 86.1%, PR: 85.0%). In the admission SOI models, including only the POA diagnosis codes, the full model and body system model also outperformed the comorbidity model.

To validate the performance of the models, we checked the prediction accuracy in the testing dataset using optimal cutoff points obtained from the training dataset as described above (Table 2). For discharge SOI, the full model had a prediction accuracy of 86.2%. The body system accuracy was 85.6%. The comorbidity model had the lowest prediction accuracy (79.3%). Similar results were observed in the admission SOI models using POA diagnosis codes.

**Table 2:** Prediction accuracy of models for predicting admission and discharge SOI

**Discharge SOI**	**Count of Variables**	**Prediction Accuracy**	**False Positive Rate**		**False Negative Rate**	
Full model	48	86.17%	6.80%		7.03%	
Comorbidity only	30	79.29%	9.79%		10.92%	
Body System CCMCC only	18	85.59%	7.28%		7.13%	
**Admission SOI**	**Count of Variables**	**Prediction Accuracy**	**False Positive Rate**		**False Negative Rate**	
Full model	48	85.41%	7.82%		6.77%	
Comorbidity only	30	77.50%	11.20%		11.30%	
Body System CCMCC only	18	84.82%	8.30%		6.88%	

We also analyzed the proportion of cases with high admission severity for our institution and other 2019-2020 U.S. News and World Report Honor Roll hospitals, by comparing 3M APR DRG SOI obtained from Vizient and the predicted results from the three models (Supplementary Fig. 1). The trends from the full model and the body system model approximated the measured APR DRG SOI. Consistent with previous results, the comorbidity model displayed the largest deviation. Although the full model and the body system model showed similar performance, the body system model exhibited greater efficiency by only including 18 predictors while the full model had 48. In summary, based on the ROC analysis, precision recall analysis, prediction accuracy and counts of predictors, the body system model was determined to be the optimal model.

**Fig. 2:**
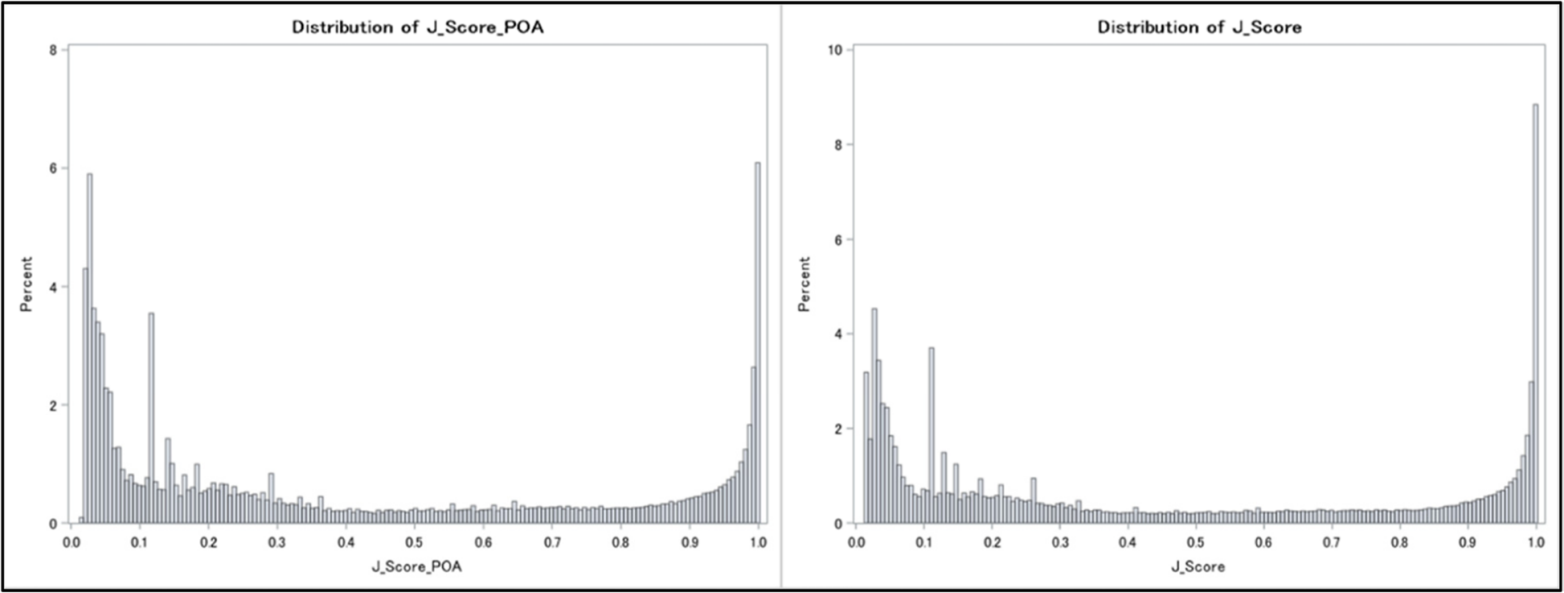
Distribution of J_Score_POA and J_Score

Besides prediction of categorical severity levels, the probability generated from the body system model also indicated the chance a case would be high severity. We referred to these probabilities as “J_Scores.” J_Scores have two versions, J_Score and J_Score_POA, depending on what diagnosis codes are used. J_Score is based on all the diagnoses coded on a case. J_Score_POA is based on only the present-on-admission (POA) and exempt diagnoses. A higher J_Score indicates more severity. To further demonstrate the J_Scores, we calculated J_Score and J_Score_POA for all the 923,266 inpatient cases, and then plotted the histograms to check their distribution (Fig. 2). As shown in Fig. 2, the histograms were bimodal with one peak at each end, indicating that the body system model performed well in separating the high and low severity cases. Notably, approximately 6.3% of the cases had a J_Score_POA of 1, and 9.1% of the cases had an overall J_Score of 1.

### Utilization of J_Scores in benchmarking and clinical documentation improvement

**Fig. 3:**
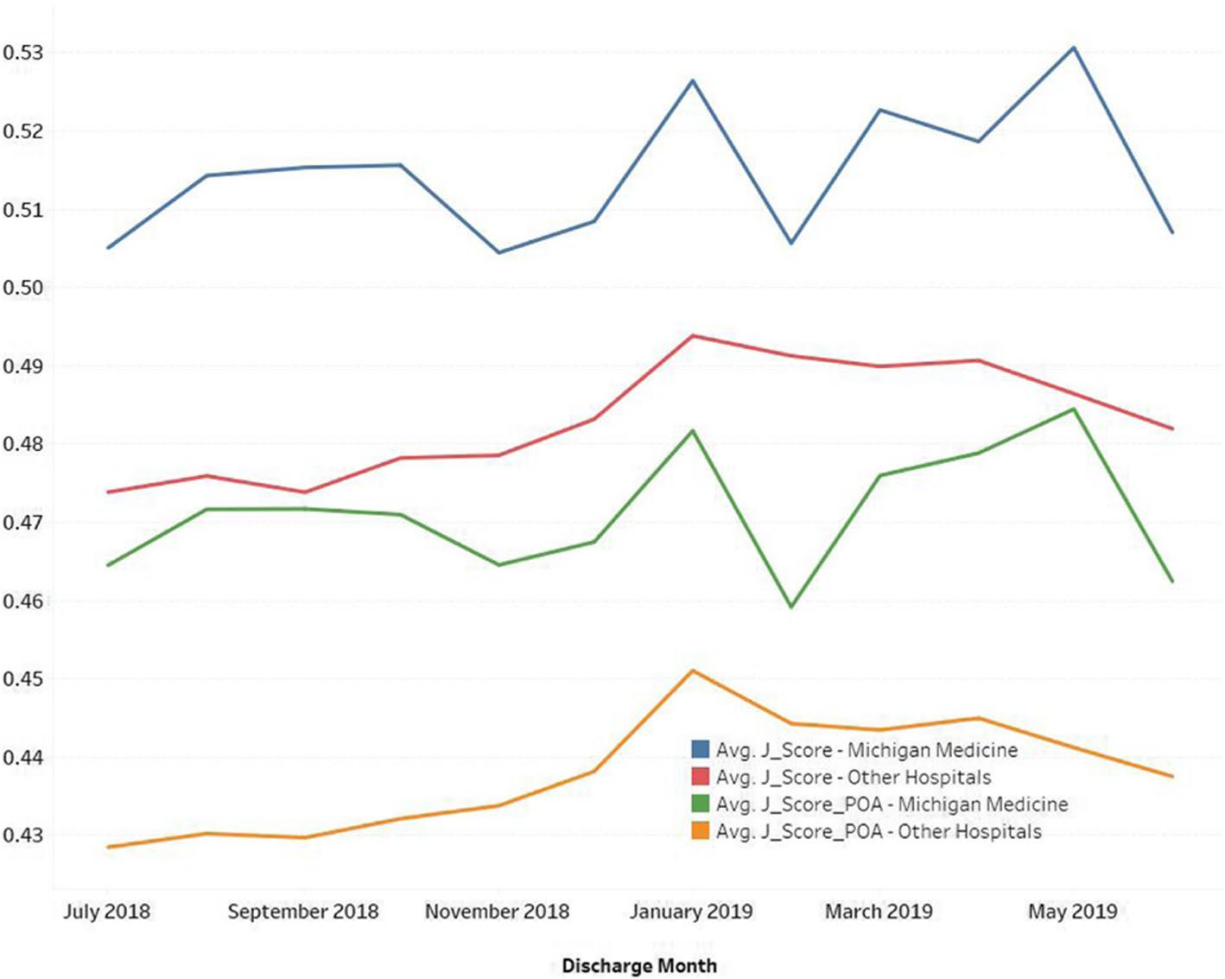
Trend of average J_Score and J_Score_POA

To further illustrate the value of J_Scores in benchmarking and CDI, we analyzed the trend of average J_Score and J_Score_POA for our institution and the other 2019–2020 US News and World Report Honor Roll hospitals (Fig. 3). Not surprisingly, the average J_Score was higher than the J_Score_POA, reflecting that more symptoms were identified, and more diagnosis codes were added after admission. The slightly increasing trend of J_Scores shows that the cases discharged in 2019 had higher severity than discharged cases in 2018. The gap between J_Score_POA and J_Score indicated non-POA problems that were found and treated during patients’ hospital stay.

**Fig. 4:**
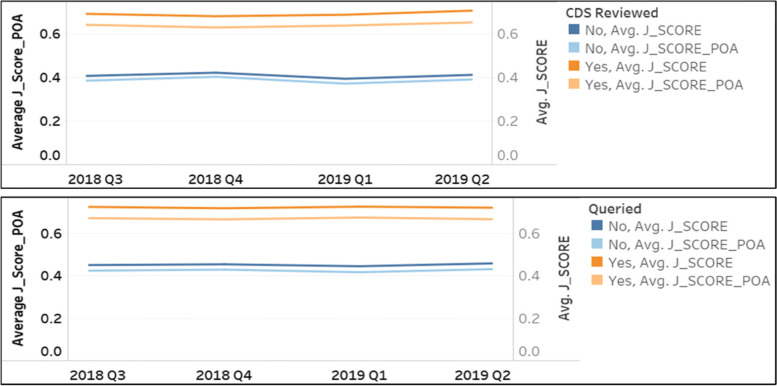
Trend of J_Score and J_Score_POA for cases reviewed or queried by clinical documentation specialists

Another application of J_Scores is to assess the performance of our Clinical Documentation Improvement program. We looked at the J_Scores for inpatient cases that had been reviewed or queried by clinical documentation specialists (CDS) (Fig. 4). Notably, the average J_Score_POA of CDS reviewed cases were much higher than non-reviewed cases, indicating that CDS were selecting cases with higher severity for review. The average J_Score was notably higher than the average of J_Score_POA for CDS reviewed or queried cases while the averages of J_Score_POA and J_Score were similar for non-reviewed or non-queried cases, suggesting that CDS activities are helpful in capturing illness severity and improving the quality of documentation.

## Discussion

In this study, we sought to develop a consistent measure of severity that is independent from the APR DRG grouper. To achieve this goal, we mapped diagnosis codes to Elixhauser comorbidities, CCI body systems, and CC/MCC indicators to create predictors and then applied orthogonal polynomial regression models to predict case severity of illness. This method can be used as an alternative way to estimate patients’ severity. We compared three models by evaluating their performance through ROC analysis, prediction accuracy, and the counts of predictors. Eventually, the body system model with CC/MCC indicators was considered the best because of higher AUC, higher accuracy rate and fewer variables. The probabilities calculated from this model were named “J_Scores,'' which serve as a measure of severity. Then, we compared the severity obtained from our model with APR DRG SOI levels acquired from the Vizient database and found that the proportions of high severity cases were similar, indicating that our model had great value in benchmarking (Supplemental Fig. 1). Plus, the large sample size of patients from 21 facilities ensured reliable results.

Although J_Scores exhibit great values in evaluating severity, the methodology differs from APR DRG SOI. The SOI subclasses developed from 3 M are determined from 3 phases with 18 steps in total after the APRDRG is assigned to a patient, incorporating the secondary diagnoses, the impact of principal diagnosis, age, OR procedure, non-OR procedures, and multiple OR procedures [31]. Each APR DRG has four subclasses of SOI. However, the severity scores generated from our model are based on affected body systems and their complication and comorbidity (CC) levels, which are not specified for APR DRG grouper. One caveat of our model is that the optimistic and promising predictive ability for severity is based on adult inpatient cases, and it needs to be validated in pediatric inpatients because pediatric cases do not have the same patterns of comorbidities as adults. Additionally, the model needs to be refreshed or re-evaluated annually to make sure it incorporates the latest version of the diagnosis codes from CMS and the updates on the body system assignment from AHRQ.

In our institution, we applied J_Scores to Vizient data to benchmark severity of illness and integrated J_Scores in CDI analysis. It was found that the average J_Score_POA of cases selected for review by CDS were much higher than the J_Score_POA of cases not selected, suggesting that our CDS were targeting cases with higher severity for review. We also envision the utility of J_Scores in audit processes, given that effective post-coding audits include a review of high-risk MS-DRGs, SOI, and risk of mortality.

## Conclusions

In this study, we developed a novel method to measure SOI using diagnoses with body system and CC/MCC indicators. It is independent from APR DRG and can be used to better evaluate or compare severity in patients from different disease categories. The results demonstrated that J_Scores generated from the body system model offer reliable predictability of patients’ illness severity on admission and at discharge. Overall, this new scoring system provides a useful tool for hospitals to benchmark SOI, assess CDI programs and direct case review to improve clinical performance and quality.

## Supplementary Information

**Additional file 1:** **Supplementary table 1.** Proportion of Elixhauser comorbidities and body system levels for training dataset and testing dataset. **Supplementary figure 1.** Proportion of cases with high severity of illness on admission.   

## Data Availability

The datasets used and/or analyzed during the current study are available from Vizient Clinical Data Base and the corresponding author on reasonable request.

## Abbreviations

AHRQ: Agency for Healthcare Research and Quality
APR DRG: All Patients Refined Diagnosis Related Groups
CC: Complication and Comorbidity
CDB: Clinical Data Base
CDI: Clinical Documentation Improvement
CMI: Case Mix Index
CMS: Center for Medicare & Medicaid Services
EROM: Expected Risk of Mortality
HCUP: Healthcare Cost and Utilization Project
LOS: Length of Stay
MCC: Major Complication and Comorbidity
MS-DRG: Medicare Severity-Diagnosis Related Groups
POA: Present-On-Admission
PR: Precision-Recall
QI: Quality Improvement
ROC: Receiver Operating Characteristic
SOI: Severity of Illness
