# Supplementary material for: Measuring case severity: a novel tool for benchmarking and clinical documentation improvement
Source: BMC Health Serv Res. 2022 Apr 15;22:513. doi: 10.1186/s12913-022-07935-1 (PMC9013032; doi:10.1186/s12913-022-07935-1)

Supplementary table 1. Proportion of Elixhauser comorbidities and body system levels for training dataset and testing dataset.

	Level	Training Dataset	Testing Dataset	P-value
**Elixhauser Comorbidity**				
CHF	No	588846 (85.04%)	196101 (84.96%)	0.36
	Yes	103604 (14.96%)	34715 (15.04%)	
VALVE	No	627185 (90.57%)	209214 (90.64%)	0.35
	Yes	65265 (9.43%)	21602 (9.36%)	
PULMCIRC	No	681265 (98.38%)	226943 (98.32%)	0.04
	Yes	11185 (1.62%)	3873 (1.68%)	
PERIVASC	No	650157 (93.90%)	216618 (93.85%)	0.45
	Yes	42293 (6.10%)	14198 (6.15%)	
HTN	No	513930 (74.22%)	171443 (74.28%)	0.58
	Yes	178520 (25.78%)	59373 (25.72%)	
HTNCX	No	681320 (98.39%)	227196 (98.43%)	0.20
	Yes	11130 (1.61%)	3620 (1.57%)	
PARA	No	661732 (95.56%)	220602 (95.57%)	0.83
	Yes	30718 (4.44%)	10214 (4.43%)	
NEURO	No	612138 (88.40%)	203905 (88.34%)	0.43
	Yes	80312 (11.60%)	26911 (11.66%)	
CHRNLUNG	No	571963 (82.60%)	190251 (82.43%)	0.06
	Yes	120487 (17.40%)	40565 (17.57%)	
DM	No	637018 (91.99%)	212200 (91.93%)	0.36
	Yes	55432 (8.01%)	18616 (8.07%)	

DMCX	No	599599 (86.59%)	199945 (86.63%)	0.68
	Yes	92851 (13.41%)	30871 (13.38%)	
HYPOTHY	No	613063 (88.53%)	204285 (88.51%)	0.70
	Yes	79387 (11.47%)	26531 (11.49%)	
RENLFAIL	No	585913 (84.61%)	195213 (84.58%)	0.65
	Yes	106537 (15.39%)	35603 (15.42%)	
LIVER	No	647167 (93.46%)	215876 (93.53%)	0.26
	Yes	45283 (6.54%)	14940 (6.47%)	
ULCER	No	684332 (98.83%)	228184 (98.86%)	0.22
	Yes	8118 (1.17%)	2632 (1.14%)	
AIDS	No	688077 (99.37%)	229337 (99.36%)	0.64
	Yes	4373 (0.63%)	1479 (0.64%)	
LYMPH	No	673970 (97.33%)	224596 (97.31%)	0.51
	Yes	18480 (2.67%)	6220 (2.69%)	
METS	No	649655 (93.82%)	216452 (93.78%)	0.46
	Yes	42795 (6.18%)	14364 (6.22%)	
TUMOR	No	620757 (89.65%)	206945 (89.66%)	0.88
	Yes	71693 (10.35%)	23871 (10.34%)	
ARTH	No	668203 (96.50%)	222767 (96.51%)	0.75
	Yes	24247 (3.50%)	8049 (3.49%)	
COAG	No	636530 (91.92%)	212104 (91.89%)	0.64
	Yes	55920 (8.08%)	18712 (8.11%)	
OBESE	No	590805 (85.32%)	196722 (85.23%)	0.28
	Yes	101645 (14.68%)	34094 (14.77%)	

WGHTLOSS	No	620925 (89.67%)	206954 (89.66%)	0.91
	Yes	71525 (10.33%)	23862 (10.34%)	
LYTES	No	523713 (75.63%)	174461 (75.58%)	0.65
	Yes	168737 (24.37%)	56355 (24.42%)	
BLDLOSS	No	672955 (97.18%)	224386 (97.21%)	0.46
	Yes	19495 (2.815%)	6430 (2.786%)	
ANEMDEF	No	570946 (82.45%)	190236 (82.42%)	0.71
	Yes	121504 (17.55%)	40580 (17.58%)	
ALCOHOL	No	667283 (96.37%)	222542 (96.42%)	0.27
	Yes	25167 (3.63%)	8274 (3.58%)	
DRUG	No	670153 (96.78%)	223349 (96.77%)	0.73
	Yes	22297 (3.22%)	7467 (3.23%)	
PSYCH	No	662402 (95.66%)	220691 (95.61%)	0.34
	Yes	30048 (4.34%)	10125 (4.39%)	
DEPRESS	No	597035 (86.22%)	199069 (86.25%)	0.76
	Yes	95415 (13.78%)	31747 (13.75%)	
Body System Indicators				
1- Infectious and parasitic diseases	0	561092 (81.03%)	186933 (80.99%)	0.77
	1	87959 (12.70%)	29320 (12.70%)	
	2	43399 (6.27%)	14563 (6.31%)	
2- Neoplasms	0	548693 (79.24%)	182876 (79.23%)	0.38
	1	68103 (9.84%)	22536 (9.76%)	

	2	75654 (10.92%)	25404 (11.01%)	
3- Endocrine; nutritional; and metabolic diseases and immunity disorders	0	263794 (38.10%)	87815 (38.05%)	0.83
	1	278041 (40.15%)	92846 (40.22%)	
	2	150615 (21.75%)	50155 (21.73%)	
4- Diseases of the blood and blood-forming organs	0	453479 (65.49%)	151177 (65.50%)	0.98
	1	145418 (21.00%)	48433 (20.98%)	
	2	93553 (13.51%)	31206 (13.52%)	
5- Mental disorders	0	444045 (64.13%)	148215 (64.21%)	0.75
	1	207090 (29.91%)	68850 (29.83%)	
	2	41315 (5.96%)	13751 (5.96%)	
6- Diseases of the nervous system and sense organs	0	410423 (59.27%)	136737 (59.24%)	0.63
	1	212015 (30.62%)	70865 (30.70%)	
	2	70012 (10.11%)	23214 (10.06%)	
7- Diseases of the circulatory system	0	292984 (42.31%)	97358 (42.18%)	0.54
	1	217933 (31.47%)	72781 (31.53%)	
	2	181533 (26.22%)	60677 (26.29%)	
8- Diseases of the respiratory system	0	475197 (68.63%)	157952 (68.43%)	0.20
	1	107128 (15.47%)	36276 (15.72%)	
	2	110125 (15.90%)	36588 (15.85%)	

9- Diseases of the digestive system	0	403359 (58.25%)	134513 (58.28%)	0.28
	1	208637 (30.13%)	69755 (30.22%)	
	2	80454 (11.62%)	26548 (11.50%)	
10- Diseases of the genitourinary system	0	457288 (66.04%)	152303 (65.98%)	0.89
	1	87238 (12.60%)	29127 (12.62%)	
	2	147924 (21.36%)	49386 (21.40%)	
11- Complications of pregnancy; childbirth; and the puerperium	0	628894 (90.82%)	209642 (90.83%)	0.54
	1	42124 (6.08%)	14122 (6.12%)	
	2	21432 (3.10%)	7052 (3.05%)	
12- Diseases of the skin and subcutaneous tissue	0	618728 (89.35%)	206096 (89.29%)	0.18
	1	46774 (6.75%)	15539 (6.73%)	
	2	26948 (3.89%)	9181 (3.98%)	
13- Diseases of the musculoskeletal system	0	495621 (71.58%)	165194 (71.57%)	0.83
	1	171189 (24.72%)	57134 (24.75%)	
	2	25640 (3.70%)	8488 (3.68%)	
14- Congenital anomalies	0	642630 (92.81%)	214367 (92.87%)	0.55
	1	23806 (3.44%)	7862 (3.41%)	
	2	26014 (3.75%)	8587 (3.72%)	

15- Certain conditions originating in the perinatal period	0	649717 (93.83%)	216698 (93.88%)	0.63
	1	34433 (4.97%)	11387 (4.93%)	
	2	8300 (1.20%)	2731 (1.18%)	
16- Symptoms, signs, and ill-defined conditions	0	388150 (56.05%)	129040 (55.90%)	0.22
	1	218708 (31.59%)	73348 (31.78%)	
	2	85592 (12.36%)	28428 (12.32%)	
17- Injury and poisoning	0	527287 (76.15%)	175717 (76.13%)	0.75
	1	116392 (16.81%)	38737 (16.78%)	
	2	48771 (7.04%)	16362 (7.09%)	
18- Factors influencing health status and contact with health services	0	84282 (12.17%)	28237 (12.23%)	0.20
	1	534097 (77.13%)	178180 (77.20%)	
	2	74071 (10.70%)	24399 (10.57%)	

Supplementary figure 1. Proportion of cases with high severity of illness on admission.

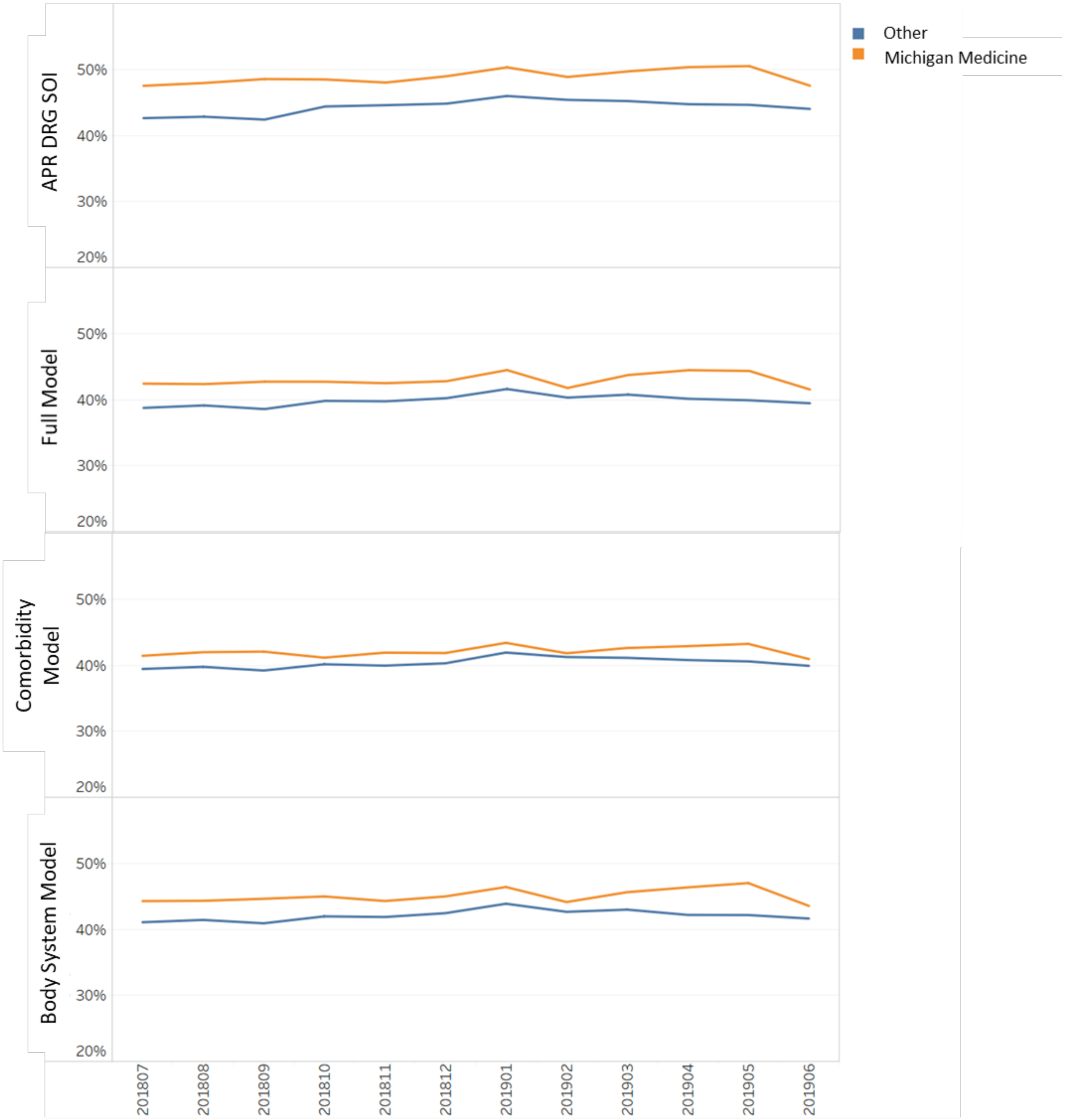

Supplement: Supplementary file 1 — Additional file 1: Supplementary table 1. Proportion of Elixhauser comorbidities and body system levels for training dataset and testing dataset. Supplementary figure 1. Proportion of cases with high severity of illness on admission. [file 12913_2022_7935_MOESM1_ESM.pdf]
